# Extracellular Signal-Regulated Kinase (ERK) Activation and Mitogen-Activated Protein Kinase Phosphatase 1 Induction by Pulsatile Gonadotropin-Releasing Hormone in Pituitary Gonadotrophs

**DOI:** 10.1155/2012/198527

**Published:** 2011-12-22

**Authors:** Haruhiko Kanasaki, Indri Purwana, Aki Oride, Tselmeg Mijiddorj, Kohji Miyazaki

**Affiliations:** Department of Obstetrics and Gynecology, Faculty of Medicine, Shimane University, Izumo 693-8501, Japan

## Abstract

The frequency of gonadotropin-releasing hormone (GnRH) pulse secreted from the hypothalamus differently regulates the expressions of gonadotropin subunit genes, luteinizing hormone **β** (LH**β**) and follicle-stimulating hormone **β** (FSH**β**), in the pituitary gonadotrophs. FSH**β** is preferentially stimulated at slower GnRH pulse frequencies, whereas LH**β** is preferentially stimulated at more rapid pulse frequencies. Several signaling pathways are activated, including mitogen-activated protein kinase (MAPK), protein kinase C, calcium influx, and calcium-calmodulin kinases, and these may be preferentially regulated under certain conditions. Previous studies demonstrated that MAPK pathways, especially the extracellular signal-regulated kinase (ERK), play an essential role for induction of gonadotropin subunit gene expression by GnRH, whereas, MAPK phosphatases (MKPs) inactivate MAPKs through dephosphorylation of threonine and/or tyrosine residues. MKPs are also induced by GnRH, and potential feedback regulation between MAPK signaling and MKPs within the GnRH signaling pathway is evident in gonadotrophs. In this paper, we reviewed and mainly focused on our observations of the pattern of ERK activation and the induction of MKP by different frequencies of GnRH stimulation.

## 1. Introduction

Reproductive functions in mammalians are regulated largely by the gonadotropins, luteinizing hormone (LH) and follicle-stimulating hormone (FSH), from anterior pituitary gonadotrophs, which are also involved in sex steroid hormone synthesis, follicular growth, and oocyte maturation [1]. Gonadotropins LH and FSH contain *α* and *β* subunits, and the *α* subunit is common to both gonadotropin hormones, whereas the *β* subunits differ from each other and confer specificity to the gonadotropin hormones [2]. Gonadotropins LH and FSH are mainly under the control of the hypothalamic peptide, gonadotropin-releasing hormone (GnRH), which is released into the hypophyseal portal vascular system [3].

 GnRH is released from hypothalamus in a pulsatile manner, in which the pulse pattern varies physiologically as a function of hormonal status and reproductive cycle stage [4, 5]. In a study using primate model, Knobil demonstrated that pulsatile secretion of GnRH, but not continuous, was essential to maintain normal LH and FSH secretion. Continuous GnRH stimulation resulted in a decrease of LH and FSH due to the downregulation of the gonadotrophs [6]. Moreover, the frequency of GnRH pulses plays a critical role in determining the output of LH and FSH from the pituitary; that is, more rapid frequencies of GnRH pulses increase the secretion of LH, whereas slower frequencies result in a decrease in LH secretion but a rise in FSH secretion [7]. In addition, changes in the frequency of GnRH pulse signals have been shown to differently regulate gonadotropin subunit gene expression. LH*β* gene expression is maximally stimulated by a GnRH pulse of 30 min interval, whereas FSH*β* gene expression is optimally stimulated by a slower GnRH pulse frequency every 2 h [8–11]. GnRH specifically controls LH and FSH secretion and also regulates LH*β* and FSH*β* subunit gene expression by changing its pattern of secretion to gonadotrophs. However, at present, how GnRH pulse frequency determines the specificity of gonadotropin subunit expression still remains unclear.

## 2. Intracellular Signal Transduction by GnRH Stimulation

**Figure 1:**
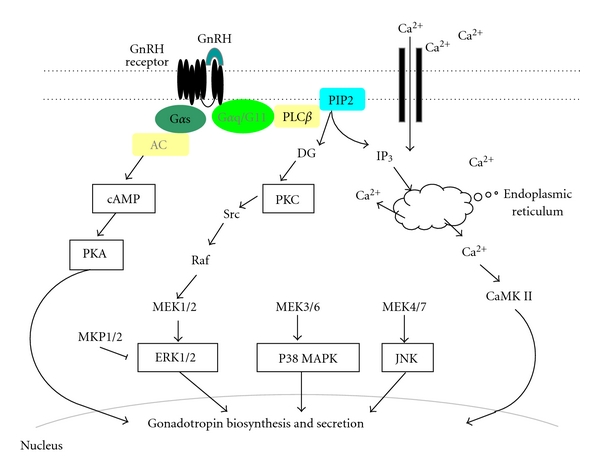
Schematic summary of intracellular signaling by GnRH. The initial phase of GnRH action involves Gq-protein-mediated stimulation of phospholipase C*β* (PLC*β*), leading to the formation of 1,4,5-triphosphate (IP3) and diacylglycerol (DG). IP3 induces the release of intracellular calcium from endoplasmic reticulum, and DG activates protein kinase C (PKC), which ultimately activates extracellular signal-regulated kinase (ERK). P38 MAPK, c-Jun N-terminal kinase (JNK), and calcium-calmodulin kinase II (CaMK II) are also activated by GnRH. Not predominant, but GnRH also couples to Gs protein which are linked with adenylate cyclase and induces rapid cAMP production, which ultimately activates protein kinase A (PKA). MAP kinase phosphatase 1/2 (MKP1/2) inactivates ERK by dephosphorylation.

The GnRH receptor is a member of seven-transmembrane G-protein-coupled receptor family. The initial phase of GnRH action involves Gq-protein-mediated stimulation of phospholipase C, leading to the formation of 1,4,5-triphosphate (IP3) and diacylglycerol (DG). Subsequently, IP3 induces the release of intracellular calcium from the endoplasmic reticulum and DG activates protein kinase C (PKC), which ultimately activates extracellular signal-regulated kinase (ERK), a member of mitogen-activated protein kinase (MAPK) family, by the activation of Raf. This activation is supported by a pathway that involves dynamin, c-Src, and Ras and a pathway that involves calcium-signaling and possibly other signaling components [12–14]. Activation of MAPK family proteins such as ERK, c-Jun N-terminal kinase (JNK), p38 MAPK, and ERK5 has been reported to mediate GnRH-induced gonadotropin subunit expression [15–19]. The JNK cascade utilizes MKK4/7 to activate transcription factors such as c-Jun, ATF2, and Elk 1 [20]. The p38 MAPK utilizes many MKK3/6 to activate Elk, AtF2, CHOP, and MEF [21]. Calcium- and calmodulin-dependent protein kinase are also activated by GnRH [22, 23]. Interestingly, the MAPK pathways are activated by the downstream of calmodulin [24]. In addition, GnRH also couples with Gs protein to increase cAMP accumulation [25]. The signal transduction systems through GnRH receptor were shown in Figure 1.

## 3. L***β***T2 Cells as a Model for Pituitary Gonadotrophs

Different types of hormone-producing cells exist in the anterior pituitary. Approximately 50% of the adenohypophysis consist of growth-hormone-producing somatotrophs and 15% are considered to be lactotrophs which produce prolactin. These cell types are acidophilic and are also believed to be derived from the same origin. Somatolactotrophs, which produce both growth hormone and prolactin, are candidate cells for the origin of lactotrophs and exist in a small population [27]. Corticotrophs, which secrete ACTH, make up approximately 15–20% and TSH-producing thyrotrophs are approximately 5% of the total adenohypophysial cell populations. Pituitary LH and FSH are secreted from gonadotrophs, and these cells likely represent up to 10% of the cell population. Although it is difficult to isolate a single colony of hormone-secreting cells, clonal strains of pituitary tumor cells have been widely used as models for the study of distinct hormone-secreting cell types. Development of the immortalized murine pituitary-gonadotroph-derived cell model, L*β*T2, has facilitated the study of signal transduction pathways activated by the GnRH receptor [28]. This cell line expresses the gonadotropin *α*-, LH*β*-, and FSH*β*-subunits as well as the GnRH receptor, and they synthesize and release LH and FSH in response to GnRH stimulation. Studies of the regulation of pituitary gonadotropin gene expression have also been performed using *α*T3-1 cells, a gonadotropin *α*-subunit-producing cell line of gonadotroph lineage [28]. Our studies were conducted using L*β*T2 cells as a model to determine the cellular response to pulsatile GnRH.

## 4. Activation of ERK by GnRH (Continuous and Pulsatile Condition) in L***β***T2 Cells

**Figure 2:**
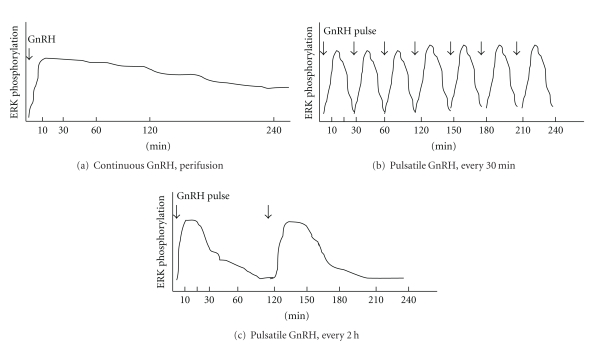
Schematic pattern of ERK activation by perifused GnRH stimulation. (a) When cells were exposed to continuous GnRH in perifused conditions, ERK activation was significantly increased, after 10 min, and increased ERK phosphorylation was sustained and gradually decreased to the basal. (b) At high frequency of GnRH pulse stimulation (one pulse very 30 min), ERK phosphorylation increased to a maximum at 10 min, and then levels rapidly decreased to bas line levels within 30 min. A similar pattern of ERK phosphorylation was observed in response to subsequent pulse of GnRH. (c) At a slower frequency of GnRH pulse stimulation (one pulse every 2 h), ERK was more rapidly phosphorylated by GnRH pulse, and increase was more sustained, remaining elevated, and then slowly decreased to the baseline levels. A similar pattern of ERK phosphorylation was observed in response to subsequent pulse of GnRH.

Generally, to study the intracellular signaling using cell culture, experiments were conducted in static condition. Cells were prepared and plated in the culture dishes and then stimulated by test reagent followed by an assay of the targets. As described above, GnRH is released in pulsatile manner in vivo, which exposes the pituitary gonadotrophs intermittently to GnRH. We examined the pattern of ERK activation induced by GnRH in static or perifused pulsatile conditions in L*β*T2 cells. In static GnRH stimulation (under which GnRH was added directly to the cell culture dish before harvesting), ERK activation was significantly increased after 10 min of GnRH stimulation and the increased ERK phosphorylation was sustained and gradually decreased to the basal level within 4–20 h [29, 30]. When cells were exposed to continuous GnRH for similar time intervals in a perifused, not static, condition, ERK phosphorylation was increased to a similar degree with maximal peak as in static culture; however, the ERK activation was more sustained and remained increased [29]. On the other hand, the ERK activation in response to pulsatile GnRH in perifused gonadotrophs was totally different compared to those observed in response to continuous GnRH. After a pulse of GnRH, ERK phosphorylation were rapidly increased, but was not sustained, returning to baseline levels within 60 min [29]. A similar pattern of ERK activation was observed in subsequent pulse of GnRH. Interestingly, the pattern of ERK phosphorylation in response to pulsatile GnRH administered at high and low frequencies were distinct. After exposures to pulsatile GnRH at high frequency (one pulse every 30 min), ERK phosphorylation increased to a maximum level at 10 min and then rapidly decreased, returning to baseline levels within 20–30 min. A similar pattern of ERK phosphorylation was observed following the next pulse of GnRH. The pattern of ERK phosphorylation in response to pulsatile GnRH at low frequency (one pulse every 2 h) was contrastingly different. By a single GnRH pulse at lower frequency, ERK was more rapidly phosphorylated, reaching a maximum level by 5 min after the pulse and remaining elevated until 20 min time point and then slowly decreasing, returning to the basal levels by 40–50 min. In light of the pattern of ERK phosphorylation during pulsatile treatment, we could speculate that ERK is activated in response to GnRH occupation of its receptor and this receptor signaling is not prolonged. It is likely that GnRH binding to its receptor has only transient effects on the downstream signaling events, which result in the unsustained ERK phosphorylation in pulsatile condition compared to the sustained pattern after continuous stimulation. In addition, the pattern of ERK phosphorylation in response to low and high GnRH pulse frequencies was distinct from each other, suggesting the role of the ERK dephosphorylation enzyme that works differently in each condition (Figure 2).

## 5. Induction of MAP Kinase Phosphatase 1 by GnRH Stimulation (Continuous and Pulsatile Condition) in L***β***T2 Cells

MAP kinase phosphatases (MKPs) are a family of protein phosphatases that inactivated MAPKs through dephosphorylation of threonine and/or tyrosine residues. MKP1 belongs to a group comprised of type I dual-specificity phosphatases (DUSPs) that inactivates ERK through dephosphorylation [31]. MKP1 is localized in the nuclear compartment, and the activation of ERK cascade is also known to promote induction of MKP1 activity, which then attenuates ERK-dependent events [32]. Therefore, MKP1 is thought to play an important role in the feedback control of ERK in the nucleus, where it attenuates the stimuli [33]. MKP1 effectively inactivates p38 MAPK and JNK [34]. With regard to the pituitary gonadotrophs, MKPs are increased in association with ERK and JNK [35, 36]. The potential feedback of regulation between MAPK signaling and MKPs with GnRH signaling pathways is also evident in gonadotrophs. In L*β*T2 cell, the induction of MKP1 occurred 60 min after GnRH stimulation and continued to be expressed in static culture, with a concomitant decrease of ERK phosphorylation [30]. MKP1 induction also occurred by pulsatile GnRH, but the expression patterns following high or low frequencies of GnRH pulse stimulation are distinct.

**Figure 3:**
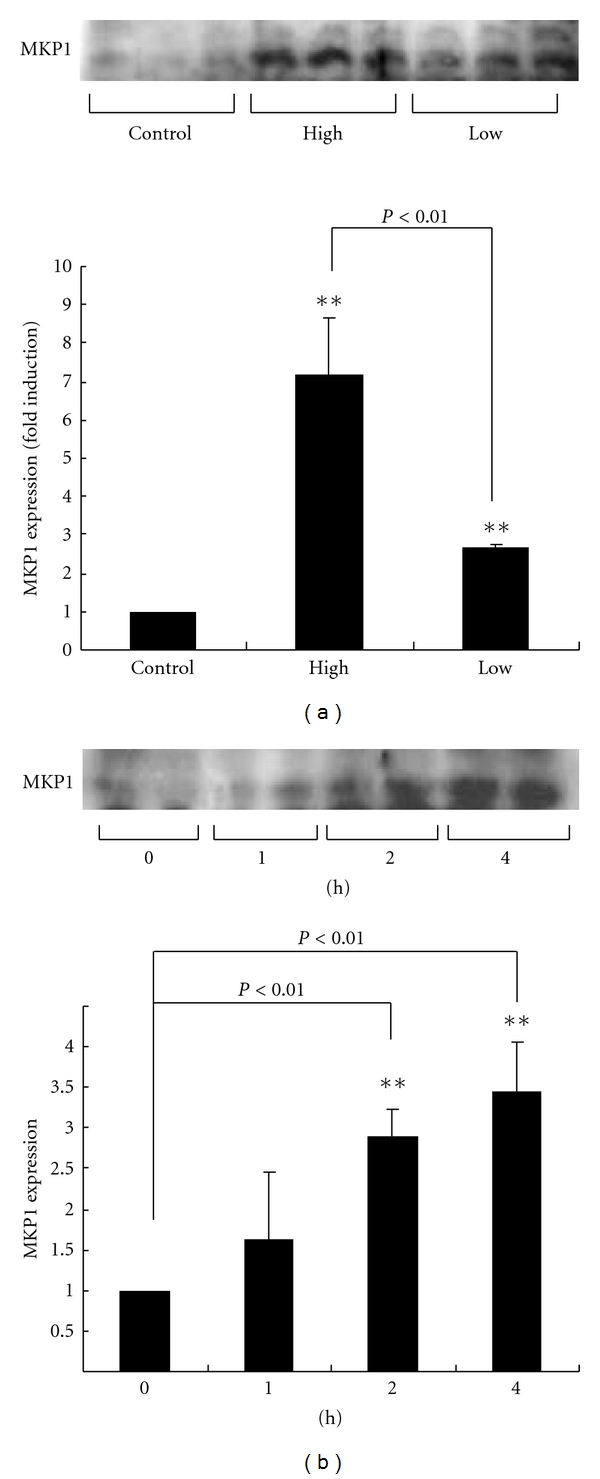
Induction of MKP1 by high frequency of GnRH pulse stimulation. (a) LbT2 cells were stimulated with vehicle (control) or pulsatile GnRH (10 nM, 5 min pulse flow per each pulse) at a frequency of one pulse every 30 min (high) or one pulse every 2 h (low). After 16 h stimulation, MKP1 protein expression was determined by Western blotting. MKP1 expression was increased predominantly following high rather than low-frequency GnRH pulse stimulation. (b) Time course of MKP1 expression following high-frequency GnRH pulse. Under the high frequency GnRH pulse stimulation, MKP1 protein expression was observed clearly at 2 h after the start of pulse stimulation (four GnRH pulses). **P < 0.01 versus control [26].

 In L*β*T2 cells, after 16 h of exposure to GnRH pulse, MKP1 expression was increased predominantly following high- (5 min GnRH pulse, every 30 min) rather than low-frequency (every 120 min) GnRH pulse stimulation. Under the high-frequency GnRH pulse stimulation, MKP1 protein expression was observed clearly at 2 h after the start of pulse stimulation (four GnRH pulses) (Figure 3). Even when greater concentration (100 nM GnRH) was applied for each pulse in low-frequency GnRH stimulation, MKP1 protein was not observed after the cells received 4 GnRH pulses [26]. These results suggest that the number of pulse and the high frequency with which they were delivered are more important than the GnRH concentration in terms of MKP1 expression induction. As described in the previous section, ERK phosphorylation occurs more rapidly, is sustained for a longer time period, and decreases more slowly in lower frequency of GnRH pulse, whereas, with high-frequency GnRH pulses, ERK phosphorylation induced by each single pulse returns to the basal level more rapidly. Another study has also shown the role of MKP1 feedback activity in modulating ERK activation and transcriptional response to GnRH [37]. These differences in the pattern of ERK phosphorylation between the various GnRH pulse stimulations might be associated with the differential regulation of MKP1 expression by pulsatile GnRH stimulation. A study by Armstrong et al. showed that the MKP had no or little effect on the rapid and transient translocation responses of ERK within nuclear level in HeLa cells, which argues against the role of MKPs or ERK-mediated feedback in shaping ERK activation during pulsatile GnRH stimulation [38].

## 6. ERK Activation and Gene Expression of Gonadotropin Subunits

The involvement of ERK pathways in GnRH-induced gonadotropin *α*-subunit [19, 39, 40], LH*β* [16, 41, 42], and FSH*β* [43] gene expressions in pituitary cells has been described in previous reports. Indeed, inhibition of ERK phosphorylation by a specific inhibitor prevented both LH*β* and FSH*β* subunit expressions dose dependently in L*β*T2 cells [30]. Overexpression of MEKK, an upstream activator of ERK, increased gonadotropin promoters without GnRH stimulation [44]. In addition, overexpression of MKPs downregulated gonadotropin promoter expression in gonadotroph cell line [30, 39]. These observations suggested that ERK pathways are strongly involved in gonadotropin subunit gene expressions.

 The pattern of ERK activation and the induction pattern of MKP1 protein were distinct in gonadotrophs stimulated by different frequencies of GnRH pulse. At present, we have not found an exact evidence on how these differences in ERK activation and induction of MKP1 contribute to the differential regulation of gonadotropin LH*β* and FSH*β* subunit gene expressions. Previously, Haisenleder et al. have reported that GnRH-stimulated FSH*β* gene expression in rat pituitary, as well as *α*-subunit and GnRH receptor, was MAPK pathway dependent, while the LH*β* gene relied on different intracellular pathways [45]. Based on these observations, we could speculate that MKP1 expression induced after high-frequency GnRH pulse might prevent FSH*β* transcription via decreased ERK activity and would affect LH*β* to a lesser extent. On the other hand, however, other studies have suggested that MAPK pathway might play a role in the GnRH regulation of LH*β* expression [46], as the transcription of LH*β* was affected to a greater extent than that of FSH*β* when ERK was inhibited [30].

## 7. Conclusion

High-frequency GnRH pulse signal from the hypothalamus stimulates the gonadotrophs to predominantly produce LH*β* subunit, whereas low-frequency GnRH pulse signal preferentially produces FSH*β* subunit. Herein, we have described the possible contribution of MAPK pathways in this regulation. There are several potential mechanisms for differential regulation of the gonadotropins LH and FSH gene. Signaling pathways stimulated by different frequencies of GnRH pulse may induce transcription factor or coactivator synthesis, cause posttranslational modifications of transcription factors, or modify the chromatin to allow transcription factors to bind, as described in a previous review [47]. In addition, other factors such as activin, follistatin, and PACAP should be considered in order to understand the mechanisms responsible for the differential regulation of LH*β* and FSH*β* by pulsatile GnRH [48–53].
